# The role of medical students in the COVID-19 pandemic response: A call for ethical guidelines

**DOI:** 10.36834/cmej.70307

**Published:** 2020-12-07

**Authors:** George T. Kitching, Adrina Zhong, Emily Kogel, Krista Wilson, Sasha Létourneau, Yipeng Ge, Céline Sayed

**Affiliations:** 1 Schulich School of Medicine and Dentistry, Western University, Ontario, Canada;; 2 School of Medicine, Queen’s University, Ontario, Canada;; 3 Faculty of Medicine, University of Ottawa, Ontario, Canada.

In the past four months, we have seen unprecedented public health measures enacted to control and mitigate the unfolding global COVID-19 pandemic. Despite these efforts, Canada’s health care system is experiencing health human resource and equipment shortages.^1^

In the face of such shortages, health care systems have recruited previously untapped resources, including reinstating retired physicians and licensing internationally-trained physicians. In March, Italy led countries in accelerating the graduation of final-year medical students to assist in the pandemic response.^2^ In Canada, medical students of all years have been quick to look for ways to contribute to the pandemic response, including voluntary childcare for essential workers in the health care system, personal protective equipment donation drives, 3-D printing, public health contact tracing, and screening at entrances to hospitals.^3^ If health human resource shortages worsen, it is reasonable to imagine Canadian medical students could be called upon to fulfill other roles in the pandemic response in order to augment the surge capacity of our health care system. However, no guidelines exist that would help protect Canada’s future doctors during such a significant role transition.

We call on government organizations, health authorities, and the medical community to develop ethical guidelines now for medical student recruitment in the event of health human resource shortages. The cost to develop these guidelines may be greater as the crisis worsens. In 2009, calls to action were made regarding the involvement of medical students in H1N1 influenza pandemic.^4^ The American Medical Association (AMA) has published basic principles for the role of medical students in responding to the COVID-19 pandemic in the United States of America.^5^ It is imperative that ethical standards for the role of Canadian medical students be articulated proactively within this public health emergency. These guidelines would allow for more rapid and efficient role modification, thus delivering effective support in a timely manner and they would serve to protect the best interests of both the students and their patients.

Medical students have a varied skillset that may be useful as demand for health human resources increases. As of May 2020, final-year students have documented competency in specific clinical skills and can function at the level of new first-year resident physicians. In comparison, first-year students, who have limited clinical skills but have had training in medical ethics and patient interviewing, can lend their skills in services such as health education. Given their basic training in infection prevention and control, students at all stages are uniquely positioned to assist staff in facilities such as long-term care homes. Many long-term care homes in Quebec and Ontario have experienced critical staff shortages in the past few weeks, leading to the deployment of Canadian forces personnel in such homes or the rapid onboarding of staff with sometimes limited health training. Given their role in curriculum development, medical school leaders are appropriately situated to assist government organizations and health authorities in defining medical student roles based on their training and skill level. Such groups could utilize these competencies to determine an appropriate level of contribution for medical students at various stages of training.

Mobilizing the medical student body as a resource to the benefit of students, patients, and the health care system requires thoughtful consideration.^6^ Similar to the AMA principles published, ethical guidelines must balance the skills students can offer with adequate supervision, patient safety, and protection of students from unnecessary risks.^5^ Specifically, these guidelines must address access to adequate training for new roles, access to sufficient PPE, deployment of students to assist in rural and remote communities, and access to adequate human resources support system, including counselling services. With many medical students contributing to the pandemic response through voluntary activities, ethical guidelines must also address issues of compensation for harm and liability inherent to volunteerism.^4^ Due to the evolving situation and limited information about COVID-19, it is difficult to create a risk assessment for medical students and their involvement in the pandemic response. While Canadian medical schools have commended the volunteerism, they emphasize that students undertake these initiatives at their own risk and without the insurance coverage typical for volunteer initiatives organized through the school. This is an impediment to medical student involvement in the pandemic response.

Medical students are eager to contribute in meaningful ways to the efforts to end the COVID-19 pandemic. However, as the demand on essential workers in the health care system increases, the potential decision to recruit medical students cannot be taken lightly. Now is the time to establish ethical guidelines for the inclusion of Canadian medical students to address the evolving needs of the health care system and public health resources resulting from this pandemic.
